# Chronic hepatitis B and metabolic dysfunction-associated steatotic liver disease: Metabolic risk factors are key drivers of hepatocellular carcinoma

**DOI:** 10.1016/j.heliyon.2024.e37990

**Published:** 2024-09-18

**Authors:** Gupse Adali, Huseyin Aykut, Nermin Mutlu Bilgic, Yusuf Yilmaz

**Affiliations:** a Department of Gastroenterology, University of Health Sciences, Istanbul Umraniye Training and Research Hospital, Istanbul, Turkiye; b Department of Gastroenterology, School of Medicine, Recep Tayyip Erdoğan University, Rize, Turkiye; c The Global NASH Council, Washington, DC, USA

**Keywords:** Chronic hepatitis B, Hepatocellular carcinoma, Metabolic dysfunction-associated steatotic liver disease, Metabolic risk factors

## Abstract

**Objective:**

Chronic hepatitis B (CHB) and metabolic dysfunction-associated steatotic liver disease (MASLD) are the leading causes of hepatocellular carcinoma (HCC). This study aimed to explore the impact of baseline MASLD on the risk of HCC development in patients with CHB receiving antiviral treatment.

**Methods:**

We consecutively recruited 535 patients with CHB who initiated antiviral treatment between January 2007 and January 2023. The exclusion criteria included coexisting HDV, HCV, or HIV infection; other chronic liver diseases; extrahepatic malignancies; prior HCC; and HCC development within one year. A baseline liver biopsy was performed in 467 patients (87 %). MASLD was defined as hepatic steatosis diagnosed histologically or by imaging, combined with one cardiometabolic risk factor. The cumulative incidence of HCC and its associated factors was analyzed in patients with CHB, with and without MASLD.

**Results:**

In total, 535 treatment-naïve patients with CHB were included, with a median follow-up of 6.05 years. MASLD was not associated with an increased incidence of HCC in patients with CHB (HR: 1.17; 95 % CI: 0.77–1.79; p = 0.466). The cumulative incidence of HCC increased with the number of fulfilled cardiometabolic criteria (0–2 criteria vs. ≥ 3 criteria) (HR: 3.93; 95 % CI: 1.89–8.19; p < 0.001).

Age (HR: 1.03, 95 % CI 1.01–1.06, p = 0.010), male sex (HR: 3.17; 95 % CI 1.34–7.53, p = 0.009), diabetes (HR: 2.81; 95 % CI 1.54–5.12, p < 0.001), and cirrhosis (HR:3.03; 95 % CI 1.57–5.5.86, p < 0.001) were independently associated with HCC development.

**Conclusions:**

It was not MASLD, but rather the presence of multiple cardiometabolic risk factors in patients with CHB that was associated with the risk of HCC in those receiving antiviral treatment. Furthermore, older age, male sex, diabetes, and cirrhosis aggravated the risk of HCC in patients with CHB.

## Highlights

• MASLD was not linked to increased HCC risk in patients with CHB on antiviral therapy.
• Multiple cardiometabolic risk factors increased HCC risk in patients with CHB.
• Older age, male sex, diabetes, and cirrhosis were independently associated with HCC development.

## 1 Introduction

Chronic hepatitis B (CHB) is a significant global health issue with a global prevalence of 3.61 % and a primary cause of hepatocellular carcinoma (HCC) [1,2]. Numerous factors increase the HCC risk in CHB patients, including a persistent high viral load, HBV genotypes, age, sex, family history, cirrhosis, diabetes, and concomitant chronic liver diseases [3]. Conversely, antiviral treatment that reduces HBV DNA levels and seroconversion from HBeAg positive to negative can lower the risk of HCC [4].

The global prevalence of non-alcoholic fatty liver disease (NAFLD) is 30 % and increasing and is the fastest growing cause of HCC [5,6]. Recently, NAFLD has been renamed as metabolic dysfunction-associated steatotic liver disease (MASLD) [7]. Although the MASLD definition may pose a slightly higher risk for mortality than the NAFLD definition due to its requirement for at least one cardiometabolic risk factor, recent evidence indicates that MASLD will likely capture the majority of patients previously defined as having NAFLD [8].

Recently, some authors have suggested that concurrent CHB and MASLD may exacerbate liver fibrosis and eventually increase the risk of HCC development [[9], [10], [11]]. However, in a recent meta-analysis of well-matched patients with CHB, steatotic liver disease was independently associated with a lower risk of HCC [12]. While antiviral treatment reduced the risk of HCC in patients with CHB, the impact of concurrent MASLD on HCC development remains unclear. This study explored whether baseline MASLD influences HCC risk in patients with CHB receiving antiviral treatment and analyzed the factors influencing HCC development.

## 2 Methods

### 2.1 Study design and patient population

**Fig. 1:**
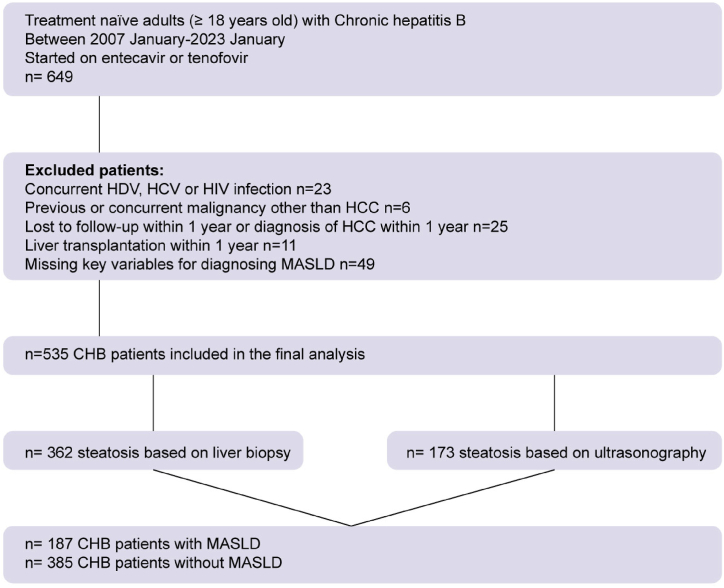
Flowchart of the study population. HDV, hepatitis D virus; HCV, hepatitis C virus; HIV, human immunodeficiency virus; HCC, hepatocellular carcinoma; MASLD, metabolic dysfunction-associated steatotic liver disease; CHB, chronic hepatitis B.

This retrospective cohort study included all HBsAg-positive patients aged ≥18 years who started antiviral treatment (n = 649) between January 2007 and January 2023 at the University of Health Sciences, Istanbul, Umraniye Training and Research Hospital, Turkey. All patients were consecutively enrolled in this study. We excluded patients with concurrent HDV, HCV, or HIV infection (n = 23); other chronic liver diseases (n = 0); previous or concurrent malignancies at baseline (n = 6); prior HCC and HCC that developed within 1 year; participants with less than 1 year of follow-up (n = 25); those who underwent liver transplantation within 1 year (n = 11); and those with missing key variables for diagnosing MASLD (n = 49) (Fig. 1). The final analysis included 535 patients with CHB.

To assess necroinflammation, fibrosis, and macrovesicular steatosis, a total of 467 (87 %) patients underwent ultrasonography-guided liver biopsy at baseline. Liver biopsy was graded according to the modified Knodell Histological Activity Index (HAI) and fibrosis was graded using the Ishak scoring system [13]. Steatosis on biopsy was defined as the presence of ≥5 % macrovesicular steatosis. Steatosis information from the liver biopsy was available for 362 patients (68 %). The diagnosis of steatosis was based on baseline ultrasonographic evaluation of patients with clinically diagnosed cirrhosis and missing histological steatosis information. Cirrhosis was diagnosed based on clinical, radiological, and/or endoscopic findings. All patients with HBV DNA >2000 IU/mL and/or at least moderate liver necroinflammation or fibrosis were started on antiviral treatment with entecavir or tenofovir [3]. HCC surveillance was performed using ultrasound and AFP every 6 months, and the diagnosis of HCC was based on the American Association for the Study of Liver Diseases guidelines [14].

This study was approved by the Institutional Ethics Committee of the University of Health Sciences Istanbul Umraniye Training and Research Hospital (11.01.2024-No:238) and followed the ethical guidelines for medical studies involving human participants outlined in the 2013 revised Declaration of Helsinki. The requirement for informed consent was waived because this study involved a retrospective review of medical records.

### 2.2 Definition of MASLD in CHB patients

MASLD was defined as the presence of hepatic steatosis identified by imaging or biopsy and no other causes of hepatic steatosis (<20 g female, <30 g male daily alcohol intake) and at least 1 out of 5 cardiometabolic criteria: BMI ≥25 kg/m^2^ or waist circumference >94 cm (male) or 80 cm (female); fasting serum glucose ≥100 mg/dL or 2-h post-load glucose levels ≥140 mg/dL or HbA1c ≥ 5.7 % or type 2 diabetes or treatment for type 2 diabetes; blood pressure ≥130/85 mmHg or specific antihypertensive drug treatment; plasma triglycerides ≥150 mg/dL or lipid lowering treatment; plasma HDL-cholesterol ≤40 mg/dL] (male) and ≤50 mg/dL (female) or lipid-lowering treatment [7].

### 2.3 Data collection

Data on baseline demographic characteristics, medical history, daily alcohol intake, history of antiviral treatment and other medications, laboratory data, follow-up period, abdominal ultrasonography findings, and liver biopsy findings (n = 467) were collected. All patients were evaluated according to the MASLD criteria and divided into two groups: CHB patients with and without MASLD. Subsequently, all cardiometabolic criteria were evaluated and categorized into groups according to the number of criteria satisfied: none, one, two, three, four, and five.

### 2.4 Statistical analysis

Continuous variables were expressed as mean ± standard deviation or median (minimum-maximum), and comparisons were conducted using the T-test or Mann–Whitney *U* test. Categorical variables were expressed as numbers and proportions, and the chi-square and Fisher's exact tests were used for comparison. A Cox proportional hazards regression model was established to determine the clinical factors independently associated with the development of HCC. Variables with p < 0.05 in univariable analyses were entered into multivariable analysis performed by Cox regression, with hazard ratios (HR) and 95 % confidence intervals (CI) calculated. Kaplan–Meier survival analysis was used to compare the cumulative incidence of HCC between CHB patients with and without MASLD, with and without steatosis, and between the number of fulfilled cardiometabolic criteria (none to two criteria vs. ≥ three criteria), and differences were tested for significance using the log-rank test. The proportional hazards assumptions were tested using the ‘cox.zph’ function in R (version 4.4.0; R Foundation for Statistical Computing, Vienna, Austria). All other statistical analyses were performed using SPSS, version 29.0.2.0 (IBM Corp., Armonk, NY, USA). Statistical significance was set at P < 0.05.

## 3 Results

### 3.1 Baseline characteristics

This study included 535 treatment-naïve chronic hepatitis B (CHB) patients with a mean age of 47 ± 13 years, of whom 63 % were male. The median follow-up period was 6.05 (1–17) years. Among them, 187 (35 %) had concurrent MASLD, 81 (15.1 %) had diabetes, 118 (22.1 %) had hypertension, 167 (31.2 %) were overweight, 136 (25.4 %) were obese, and 190 (35.5 %) had cirrhosis. The distribution of fibrosis stages was as follows: F0 (n = 13, 2.8 %), F1 (n = 36, 7.7 %), F2 (n = 135, 28.9 %), F3 (n = 110, 23.6 %), F4 (n = 68, 14.6 %), F5 (n = 28, 6 %), and F6 (n = 77, 16.5 %). Liver biopsy revealed steatosis in 153 patients (28.6 %). The percentage of patients who were HBeAg positive was 18.1 % (n = 97). Regarding antiviral treatment, 145 patients (27 %) were on entecavir and 390 patients (73 %) were on tenofovir. The numbers of patients with 0, 1, 2, and ≥3 cardiometabolic risk factors were 147 (27.5 %), 236 (44.1 %), 114 (21.3 %), and 38 (7.1 %), respectively.

Patients with concurrent MASLD were older; had higher BMI; had diabetes, hypertension, cirrhosis, and steatosis on liver biopsy; and lower HBV DNA levels than those without MASLD. The baseline characteristics of the CHB patients with and without MASLD are illustrated in Table 1.

**Table 1:** Baseline characteristics of patients with chronic hepatitis B with and without MASLD.

Characteristic	Number	Without MASLD n = 348	With MASLD n = 187	p
**Age (years)**	535	45.96 ± 14.38	49.87 ± 10.69	<0.001
**Age of HCC diagnosis**	46	63.2 ± 12.7	60.9 ± 11	0.538
**Sex, male**	535	219 (62.9)	119 (63.6)	0.872
**BMI, kg/m**^**2**^	434	25.1 (16.3–44.1)	30.1 (51.9–30.6)	<0.001
**Diabetes**	535	28 (8)	53 (28.3)	<0.001
**Hypertension**	535	58 (16.7)	60 (32.1)	<0.001
**Triglyceride ≥ 150 mg/dL**	531	26 (7.5)	21 (11.3)	0.146
**HDL-cholesterol, mg/dL** **≤ 40 (M) or ≤ 50 (F)**	273	28 (16.4)	16 (15.7)	0.881
**Cirrhosis**	535	134 (38.5)	56 (29.9)	0.049
**Significant fibrosis (≥ F2)**	467	264 (90.7)	154 (87.5)	0.271
**Steatosis on biopsy**	362	20 (9.3)	133 (89.9)	<0.001
**HBV DNA, IU/mL**	534	442,545 (10–17 × 10^8^)	77,489 (75–797142790)	0.007
**HBeAg seropositivity**	498	72 (22)	25 (14.7)	0.053
**Glucose, mg/dL**	438	90 (59–318)	95 (71–254)	0.003
**ALT, U/L**	513	43 (9–281)	39 (10–285)	0.095
**HCC**	535	29 (8.3)	17 (9.1)	0.766

Data are expressed as medians (minimum-maximum) or numbers (%).

MASLD, metabolic dysfunction-associated steatotic liver disease; BMI, body mass index; HDL, high-density lipoprotein; HBV, hepatitis B virus; HCC, hepatocellular carcinoma.

The median follow-up duration for patients with CHB who developed HCC was 5.4 years (1.3–14 years). The mean age at HCC diagnosis was 62.4 ± 12 years. When comparing CHB patients with and without MASLD, the median follow-up duration for those who developed HCC was 5.4 years (1.8–14 years) in patients without MASLD, and 6.5 years (1.3–12.3 years) in patients with MASLD (p = 0.577). The mean age at HCC diagnosis was 63.2 ± 12.7 years in patients without MASLD and 60.9 ± 11 years in patients with MASLD (p = 0.538) (Table 1).

### 3.2 HCC incidence and risk factors

During the follow-up period, 46 patients with CHB (8.6 %) were diagnosed with HCC: 17 (9.1 %) with concurrent MASLD and 29 (8.3 %) without MASLD. The 1-year, 3-year, 5-year, 10-year, and 15-year cumulative incidences of HCC were 0.6 %, 2.4 %, 4.1 %, 6.9 %, and 8.6 %, respectively. The 15-year cumulative incidence of HCC was 8.3 % in patients without MASLD and 9.1 % in those with MASLD. Among patients with 0, 1, 2, and ≥3 cardiometabolic risk factors, the corresponding 15-year cumulative incidences of HCC were 6.1 %, 7.6 %, 8.8 %, and 23.6 %, respectively.

**Fig. 2:**
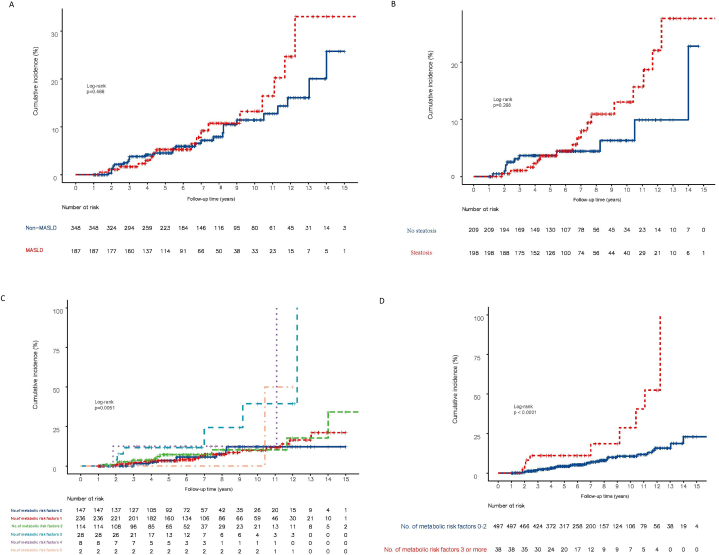
Kaplan-Meier estimates of the cumulative incidence of HCC in CHB patients (A) with and without MASLD (B) with and without steatosis (C) stratified by the number of metabolic risk factors (0, 1, 2, 3, 4, 5) and (D) stratified by the number of metabolic Risk Factors (0–2 vs. ≥3).

The cumulative incidence of HCC was not significantly different between CHB patients with and without MASLD (aHR: 1.17; 95 % CI: 0.77–1.79; p = 0.466) and in patients with and without steatosis (aHR: 1.53; 95 % CI: 0.72–3.27; p = 0.273) (Fig. 2A and B). Patients with CHB were classified according to the number of patients who fulfilled the cardiometabolic criteria. The cumulative incidence of HCC increased with an incline in the number of cardiometabolic risk factors (p = 0.0051). Compared with patients without cardiometabolic risk factors, those with one risk factor had an aHR: 0.52 (95 % CI 0.17–1.61, p = 0.258), those with two risk factors had an aHR: 4.57 (95 % CI 1.18–17.68, p = 0.027), those with three risk factors had an aHR: 0.77 (95 % CI 0.23–2.62, p = 0.674), those with four risk factors had an aHR: 0.97 (95 % CI 0.36–2.62, p = 0.952), and those with five risk factors had an aHR: 1.28 95 % CI: 0.58–2.86, p = 0.540) (Fig. 2C). Patients with three or more cardiometabolic criteria had a significantly higher HCC incidence (aHR: 3.93; 95 % CI: 1.89–8.19; p < 0.001) compared to patients with 0–2 cardiometabolic criteria (Fig. 2D).

In the univariate analysis, age, male sex, cirrhosis, diabetes, hypertension, and ≥3 cardiometabolic risk factors (compared to 0–2 criteria) were significantly associated with the development of HCC (Table 2). In the multivariable Cox regression model, diabetes (HR: 2.81; 95 % CI 1.54–5.12, p < 0.001), cirrhosis (HR: 3.03; 95 % CI 1.57–5.5.86, p < 0.001), age (HR: 1.03, 95 % CI 1.01–1.06, p = 0.010), and male sex (HR: 3.17; 95 % CI 1.34–7.53, p = 0.009) were significantly associated with HCC occurrence. (Table 3).

**Table 2:** Univariable analysis of factors associated with HCC development in patients with chronic hepatitis B.

Variables	Hazard Ratio	95 % CI	p
**Age (years)**	1.07	1.05–1.10	<0.001
**Sex, male**	4.00	1.69–9.44	0.002
**BMI, kg/m**^**2**^	1.05	0.99–1.10	0.061
**Diabetes**	3.96	2.2–7.14	<0.001
**Hypertension**	2.35	1.28–4.32	0.006
**Cirrhosis**	4.56	2.40–8.68	<0.001
**HBV DNA, IU/mL**	1.00	1.00–1.00	0.092
**HBeAg seropositivity**	1.20	0.53–2.69	0.660
**Steatosis**	1.53	0.72–3.27	0.273
**MASLD**	1.25	0.69–2.28	0.465
**Cardiometabolic risk factors 0**–**2 vs ≥ 3 criteria**	3.93	1.89–8.18	<0.001

HCC, hepatocellular carcinoma; CI, confidence interval; BMI, body mass index; HBV, hepatitis B virus; MASLD, metabolic dysfunction-associated steatotic liver disease.

**Table 3:** Multivariable analysis of factors associated with HCC development in patients with chronic hepatitis B.

Variables	Hazard Ratio	95 % CI	p
**Diabetes**	2.81	(1.54–5.12)	<0.001
**Cirrhosis**	3.03	(1.57–5.86)	<0.001
**Sex, male**	3.17	(1.34–7.53)	0.009
**Age (years)**	1.03	(1.01–1.06)	0.010

HCC, hepatocellular carcinoma; CI, confidence interval.

## 4 Discussion

This study showed that the baseline MASLD and hepatic steatosis were not associated with an increased risk of HCC in patients with CHB receiving antiviral treatment. Instead, the presence of multiple cardiometabolic risk factors was associated with an increased risk of developing HCC. Furthermore, traditional risk factors such as age, male sex, diabetes, and cirrhosis were independently associated with the occurrence of HCC. These findings support the notion that metabolic risk factors are more important contributors to HCC development than steatosis alone.

Multiple conflicting findings have emerged regarding the influence of steatosis on the risk of HCC in patients with CHB. Studies have shown that steatosis increases the risk of HCC in patients with CHB [[9], [10], [11],[15], [16], [17]]. However, other studies have not confirmed this association [[18], [19], [20], [21], [22], [23], [24]]. Instead, some have reported that concurrent steatosis decreases the risk of HCC development [[25], [26], [27], [28]]. Majority of the studies reported that steatosis increased HCC incidence and diagnosed steatosis via liver biopsy.^9,11, 16,17^Additionally, two recent meta-analyses, including all these studies, demonstrated that biopsy-proven steatosis in patients with CHB was associated with an increased risk of HCC development. Mao et al. analyzed 16 studies involving 60,213 patients and found that biopsy-proven steatosis was significantly associated with the risk of HCC [29]. Wong et al. included 11 studies involving 14,014 patients who provided outcome data for HCC in patients with CHB. Pooled data revealed a protective effect against steatosis in patients with CHB. However, a significantly higher incidence of HCC was found in patients with biopsy-diagnosed steatosis than in those without [12]. The majority of our study cohort also underwent liver biopsy, all patients received antiviral treatment throughout the follow-up period, and there was no association between steatosis and HCC development. This result could be attributed to the different biopsy indications compared with other studies. We performed biopsies of all subjects with HBV DNA >2000 IU/mL, except for clinically diagnosed patients with cirrhosis, to initiate antiviral treatment. Consequently, in our cohort, there was no difference in the significant fibrosis (F ≥ 2) rates between patients with and without MASLD. Conversely, biopsy cohorts from other studies were more likely to have advanced fibrosis, cirrhosis, elevated transaminase levels, or significant fibrosis detected by noninvasive tests, indicating potential selection bias in those studies [11,27]. Moreover, in our study, the majority of patients with HCC (n = 33, 72 %) were cirrhotic at baseline, and steatosis is known to decrease in cirrhosis. Another rationale for failure of finding an association between steatosis and HCC development in or study could be the “burn-out” of hepatic steatosis during fibrosis progression.

Recent changes in the nomenclature from NAFLD to MASLD have led to studies including metabolic dysfunction and steatosis as risk factors for HCC development in patients with CHB. Van Kleef et al. showed that metabolic dysfunction-associated fatty liver disease (MAFLD) was associated with reduced HCC-free survival in patients with CHB and that concomitant steatohepatitis did not increase the risk of HCC. Furthermore, in 14 % of patients with steatosis who did not satisfy the MAFLD criteria, there was no increased risk of adverse outcomes compared with patients with CHB without steatosis [11]. Yu et al. and Huang et al. found that MAFLD was associated with a decreased risk of HCC development; however, metabolic dysfunction increased the risk of HCC [23,28]. Consistent with previous studies, Patmore et al. showed that metabolic comorbidities such as diabetes mellitus, hypertension, dyslipidemia, and being overweight in patients with CHB were associated with an increased risk of HCC development, and the risk was notably higher in patients with multiple comorbidities [30]. Song et al. reported that concomitant CHB with MAFLD was not associated with an increased risk of HCC [24]. Our study revealed no association between the risk of HCC in patients with CHB infection and concurrent MASLD. This is the first study to assess the association between the risk of HCC and concurrent CHB and MASLD, which is characterized by hepatic steatosis and one or more cardiometabolic criteria. MAFLD differs from MASLD in that it requires being overweight or obese, having type 2 diabetes, or having metabolic dysfunction [31]. The MAFLD criteria may present a slightly higher risk of HCC development, which could explain our finding of no association between the MASLD and the risk of HCC. However, when we examined the cardiometabolic risk factors in relation to HCC development, we found that the HCC risk increased with the number of these factors. Notably, patients with three or more metabolic risk factors at baseline had a 3.93-fold higher risk of developing HCC than those with 0–2 metabolic risk factors. This finding is consistent with those of previous studies that have shown an increased number of metabolic risk factors that increase the risk of HCC development in patients with CHB [11,23,28,30,32]. These findings suggests that, rather than MASLD or hepatic steatosis, it is the metabolic risk factors that predominantly drive the risk of HCC in patients with CHB.

Diabetes is a recognized risk factor for HCC and is one of the metabolic risk factors included in the definition of MASLD. Our study confirmed that diabetes is an independent risk factor for HCC, which is consistent with existing evidence [33]. Furthermore, older age, male sex, and baseline cirrhosis were independently associated with the incidence of HCC, all of which are established risk factors for HCC. Our study aligns with the existing literature, demonstrating that traditional metabolic risk factors, rather than hepatic steatosis alone, significantly contribute to the risk of HCC development in patients with CHB. Despite the lack of an association between MASLD and HCC in our cohort, the presence of multiple cardiometabolic risk factors significantly increased the risk of HCC. These findings highlight the need to address metabolic comorbidities in the management of patients with CHB.

Although this study featured an unbiased biopsy cohort with a long follow-up period of >15 years, patients treated with antivirals, a sufficient number of HCC events, and well-defined cardiometabolic risk factors, several limitations must be acknowledged. First, it was a retrospective cohort study with a relatively small sample size and was conducted at a single tertiary center. Second, only patients who were cirrhotic or had an HBV DNA >2000 IU/mL and either a fibrosis stage >1 or HAI score >5 were initiated on antiviral treatment. These exclusion criteria may have omitted patients with mild fibrosis, low necroinflammatory activity, and inactive carriers, potentially limiting the representation of the entire spectrum of patients with CHB. Third, liver biopsies were not evaluated for nonalcoholic steatohepatitis (NASH)/metabolic-associated steatohepatitis (MASH), which would have enabled the analysis of the impact of MASH on HCC development. Fourth, cardiometabolic risk factors were recorded only at baseline, and patients who developed these risk factors during the follow-up period were not included in the final analysis.

In conclusion, our study underscores the predominance of metabolic risk factors for hepatic steatosis in driving the risk of HCC among patients with CHB undergoing antiviral treatment. These findings suggest that the management of patients with CHB should focus on early detection and management of metabolic risk factors to mitigate the risk of HCC. Future prospective studies exploring the impact of MASH on HCC development with standardized diagnostic criteria and longitudinal designs are essential to refine risk stratification and improve clinical outcomes in this patient population.

## Funding statement

The study was supported by the Recep Tayyip Erdoğan University Development Foundation (Grant number: 02024007024023).

## CRediT authorship contribution statement

**Gupse Adali:** Writing – review & editing, Writing – original draft, Methodology, Conceptualization. **Huseyin Aykut:** Visualization, Formal analysis. **Nermin Mutlu Bilgic:** Data curation. **Yusuf Yilmaz:** Writing – review & editing, Supervision, Methodology, Funding acquisition, Conceptualization.

## Declaration of competing interest

The authors declare the following financial interests/personal relationships which may be considered as potential competing interests: The authors declare the following financial interests/personal relationships which may be considered as potential competing interests: The corresponding author, Yusuf Yilmaz, is an associate editor of this journal. If there are other authors, they declare that they have no known competing financial interests or personal relationships that could have appeared to influence the work reported in this paper. If there are other authors, they declare that they have no known competing financial interests or personal relationships that could have appeared to influence the work reported in this paper.
