# Supplementary material for: Chronic hepatitis B and metabolic dysfunction-associated steatotic liver disease: Metabolic risk factors are key drivers of hepatocellular carcinoma
Source: Heliyon. 2024 Sep 18;10(18):e37990. doi: 10.1016/j.heliyon.2024.e37990 (PMC11425165; doi:10.1016/j.heliyon.2024.e37990)
Supplement: Multimedia component 1 [file mmc1.docx]

The Supplementary Table 1 presents the results of the proportional hazards assumptions test using Schoenfeld residuals for each predictor and the overall model. For Age variable the chi-square value is 0.5858 with 1 degree of freedom (df) and a p-value of 0.44. This suggests that the proportional hazards assumption holds for age, as the p-value is not significant (typically, a p-value > 0.05 indicates no violation of the assumption). For male sex the chi-square value is 0.0497 with 1 df and a p-value of 0.82. This indicates that the proportional hazards assumption holds for male sex. For diabetes the chi-square value is 1.8897 with 1 df and a p-value of 0.17. This suggests that the proportional hazards assumption holds for diabetes. For cirrhosis The chi-square value is 0.7518 with 1 df and a p-value of 0.39. This indicates that the proportional hazards assumption holds for cirrhosis. For GLOBAL (overall model) the overall chi-square value is 3.7176 with 4 df and a p-value of 0.45, this suggests that the proportional hazards assumption holds for the overall model.

**Suppl. Table 1: Proportional hazards assumptions using Schoenfeld residual for each predictor and overall model.**

|  | **Chi-square** | **df** | **p** |
| --- | --- | --- | --- |
| Age | 0.5858 | 1 | 0.44 |
| Male sex | 0.0497 | 1 | 0.82 |
| Diabetes | 1.8897 | 1 | 0.17 |
| Cirrhosis | 0.7518 | 1 | 0.39 |
| GLOBAL | 3.7176 | 4 | 0.45 |

The plot shows that the horizontal red line (no change) for age, male sex, diabetes, and cirrhosis shows most of the time fall in between 95% confidence interval which indicates that there is no change of hazard ration for these variables, so the assumption of cox regression hazard proportional assumptions seems to be fulfilled.


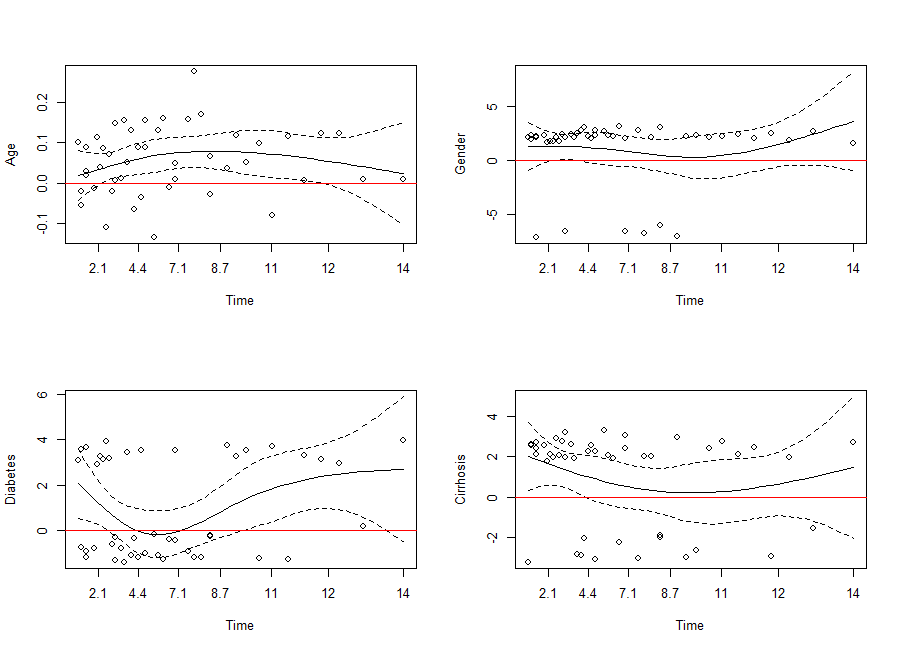


**Suppl. Figure** **1:** Plot of Schoenfeld residuals for each predictor over time.
